# P-587. Durable Virological Suppression, Reduced Body Weight, and Improved Serum Cholesterol after Switch to Tenofovir DF-Containing, Ainuovirine-Based Antiretroviral Regimen in People with *HIV-1*: the 96-Week Results of the SPRINT Trial, a Randomized, Active-Controlled Phase 3 Extensional Study

**DOI:** 10.1093/ofid/ofae631.785

**Published:** 2025-01-29

**Authors:** Fujie Zhang, Hao Wu, ping ma, Qingxia Zhao, hongxia wei, Hongzhou Lu, Hui Wang, Shenghua He, Zhu Chen, yaokai Chen, Ming wang, Weiping Cai, Hong Qin

**Affiliations:** Beijing Ditan Hospital, Beijing, Beijing, China; Beijing Youan Hospital Affiliated to Capital Medical University, Beijing, Beijing, China; Nankai University Second People's Hospital, School of Medicine, Nankai University, tianjin, Tianjin, China; Zhengzhou Municipal Sixth People’s Hospital and Infectious Disease Hospital of Henan Province, Zhengzhou, Henan, China; The Second Hospital of Nanjing, Nanjing University of Chinese Medicine, Nanjing, Jiangsu, China; Shenzhen Municipal Third Hospital, Shenzhen, Guangdong, China; Shenzhen Municipal Third Hospital, Shenzhen, Guangdong, China; Chengdu Municipal Public Health Clinical Center, Chengdu, Sichuan, China; Chengdu Municipal Public Health Clinical Center, Chengdu, Sichuan, China; Chongqing Public Health Medical Center, chongqing, Chongqing, China; The First Hospital of Changsha, Changsha, Hunan, China; Guangzhou Municipal Eighth People’s Hospital Affiliated to Guangzhou Medical University, Guangzhou, Guangdong, China; Jiangsu Aidea Pharmaceutical Co., Ltd, Yangzhou, Jiangsu, China (People's Republic)

## Abstract

**Background:**

The SPRINT trial was a multi-center, randomized, double-blind, active-controlled study in virologically suppressed people with HIV-1 (PWHs) switching from efavirenz-based antiretroviral (ARV) regimen. This study compared the efficacy and safety profiles of switch to tenofovir DF (TDF) containing, ainuovirine (ANV) based regimen (ACC008), and that to tenofovir alafenamide (TAF) containing, boosted elvitegravir (EVG/c) based regimen. ACC008 was shown to be non-inferior in virological efficacy but with reduced weight gain and improved lipid profile compared to the comparator at 48 weeks. We herein reported the week 48 to 96 results of the extensional study period as prespecified.

**Figure 1.**
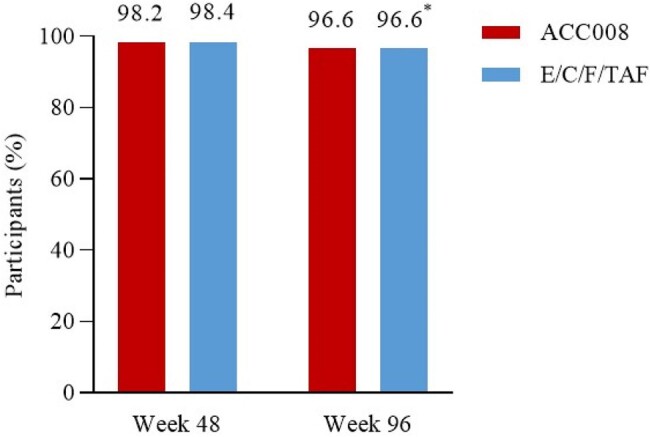
Durable virological suppression after switch to ACC008 from efavrienz- and boosted elvitegravir-based antiretroviral regimens.

Snapshot analysis of the proportion of participant with HIV-1 RNA<50 copies/mL. *Switching to ACC008 after 48 weeks of E/C/F/TAF treatment (mITT-E). ACC008, ainuovirine/ lamivudine/ tenofovir disoproxil; E/C/F/TAF, elvitegravir/ cobicistat/ emtricitabine/ tenofovir alafenamide. mITTE, modified intention-to-treat-exposed.

**Methods:**

Out of 762 adult PWHs randomized in the SPRINT trial, 755 PWHs continued the extensional study, and all received open-label ACC008 for additional 48 weeks (ACC008→ACC008 arm, n=378; comparator→ACC008 arm, n=377). The primary efficacy endpoint was the proportion of PWHs with plasma HIV-1 RNA below 50 copies/mL at week 96 as per the snapshot algorithm. Primary safety outcomes of interest included changes in body weight, and serum low-density lipoprotein cholesterol (LDL-C) at week 96 from week 48.

**Figure 2.**
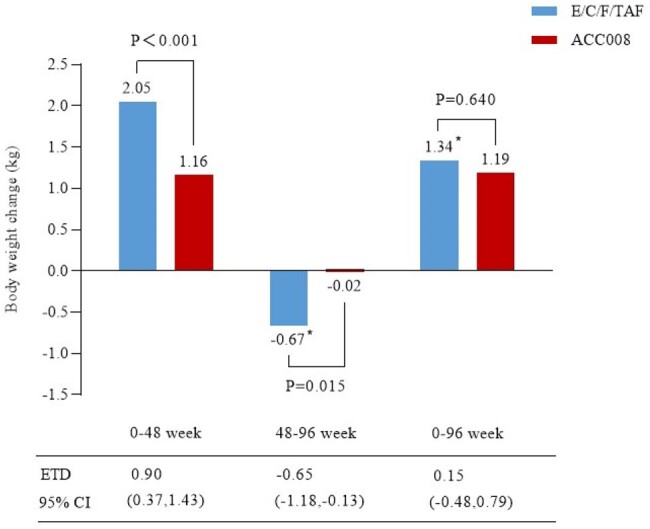
Significant weight loss after switch to ACC008 from E/C/F/TAF at weeks 48-96. *Switching to ACC008 after 48 weeks of E/C/F/TAF treatment. ACC008, ainuovirine/ lamivudine/ tenofovir disoproxil; E/C/F/TAF, elvitegravir/ cobicistat/ emtricitabine/ tenofovir alafenamide.

**Results:**

At 96 weeks, 96.6% (368/381) of PWHs maintained virological suppression in ACC008→ACC008 arm as per the intention-to-treat-exposed analysis (ITT-E, n=381); 96.6% (364/377) of PWHs remained virologically suppressed in comparator→ACC008 arm as per the modified ITT-E analysis (mITT-E, n=377). Changes in body weight (least square mean) from week 48 were -0.67 kg for comparator→ACC008 arm, and -0.02 kg for ACC008→ACC008 arm (estimated treatment difference [95%CI], -0.65 kg [-1.18, -0.13], p=0.015) at week 96, respectively. Changes in serum LDL-C from week 48 were -0.24 mmol/L, and -0.05 mmol/L (-0.19 mmol/L [-0.34, -0.03], p=0.019) at week 96, respectively.

**Figure 3.**
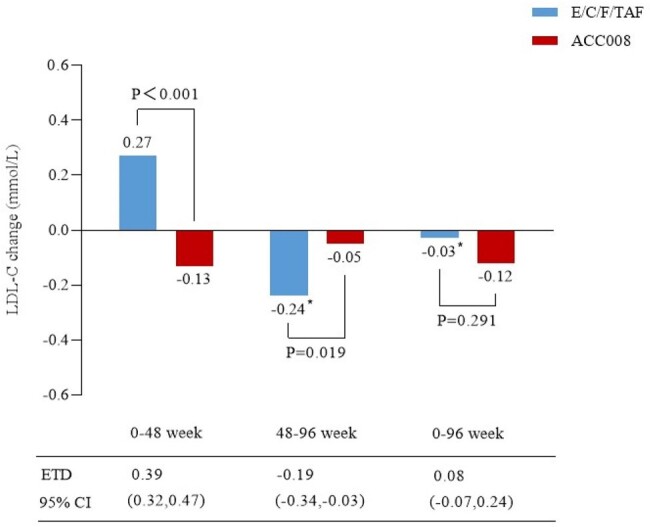
Significant LDL-C decline after switch to ACC008 from E/C/F/TAF at weeks 48-96. *Switching to ACC008 after 48 weeks of E/C/F/TAF treatment. ACC008, ainuovirine/ lamivudine/ tenofovir disoproxil; E/C/F/TAF, elvitegravir/ cobicistat/emtricitabine/ tenofovir alafenamide; LDL-C, low-density lipoprotein cholesterol.

**Conclusion:**

Switch to tenofovir DF-containing, ANV-based regimen maintained virological suppression, and that from TAF-containing, EVG/c-based regimen also resulted in high suppression at week 98. Additional benefits included weight loss and serum LDL-C decline after switch to tenofovir DF-containing, ANV-based from TAF-containing, EVG/c-based regimen.

**Disclosures:**

**Hong Qin, MD, PhD**, Jiangsu Aidea Pharmaceutical Co., Ltd: Honoraria

